# Notes and News

**Published:** 1893-01-07

**Authors:** 


### NOTES AND NEWS.

COLLECTED AND COMMUNICATED.

THE French Minister of the Interior has been authorised to formulate a project for rewarding the services of those who have especially distinguished themselves in the late cholera epidemic. The rewards are to take the form of decorations of three classes.

THE Poor Law Commission about to sit to consider whether any alterations in the system of poor law relief are desirable in the case of persons whose destitution is occasioned by incapacity for work, resulting from old age, and the various bearings of these cases, includes the names of the Prince of Wales, Lord Lingen, Lord Playfair, Mr. James Stuart, Mr. Charles Booth, Mr C. S. Loch, and twelve others.

A PRIZE of 3,000 francs has been offered by Baron Leon de Lenval, of Nice, to the inventor of the best application of the principles of the microphone in the construction of a portable apparatus for the improvement of the hearing of deaf persons. The competition closed on the last day of the old year, and we have yet to learn whether there has been any useful outcome. Such an invention would be most valuable, and the inventor would earn the gratitude of countless numbers of afflicted persons.

AS a revival on modern lines of a religious and charitable institution, St. Leonard's Hospital, at Bedford, has been opened for convalescents. A picturesque exterior has been chosen, and the hospital is after the fashion of the old gabled houses. The original intention of the founders is to be adhered to, and the object, therefore, will mainly be to provide good diets to convalescents in their own homes from the institution, and to send patients to convalescent homes. An excellent reading-room and library is attached to the hospital, as it is called, for the use of the poor. It will be seen that this St. Leonard's Hospital is a benevolent institution therefore, and not a medical charity.

MONSIEUR PASTEUR has had honours paid him by several foreign countries on the occasion of his seventieth birthday on the 27th ult. The Medical Society of Sweden delegated their President, Dr. Erik Nordenson to present to Mons. Pasteur, with the concurrence of King Oscar, a gold medal cast in honour of the savant. The Medical Society of Berlin has elected him an honorary member, and the University of Geneva has conferred upon him the honorary title of Doctor of Medicine of the University. Sir Joseph Lister represented English Medical Science, and the Royal Society in particular, and presented an address written by the President of the Society. This seventieth anniversary of an honoured life was made the occasion of the utmost demonstration of esteem on the part of Mons. Pasteur's own countrymen. Mons. Carnot himself occupied the chair at the great meeting assembled to do honour to the veteran. Mons. d'Abbardie, President of the Academy of Science, presented him with the gold medal, especially struck in his honour, and bearing the inscription on one side: "A Pasteur, le jour de ses Soixante-dix Ans, la Science et l'humanité reconnaiss autes, 27 Décembre, 1892." (To Pasteur, on his seventieth birthday, in token of recognition from science and humanity.)

THERE is a growing feeling amongst humanitarians that some sort of supervising care should be exercised towards the weak-minded of the population. The Society for the Protection of Children has proved most beneficial. Weak-minded persons are still more in need of friendly assistance than children, from the fact that they are frequently regarded as life-long burdens to those connected with them. The case of the peasant woman in Austria, who kept her weak-minded step-daughter imprisoned for three years and a half, may be a very unique instance of such treatment, owing to the difficulty attending such a procedure, but cruelty towards these helpless beings is only too frequently exercised to be denied. Daily in our towns and in our villages we may meet with these unfortunate creatures, who, dull of intellect in the first instance, have relapsed into hopeless imbeciles, the prey and butt of the ever-irrepressible urchin, when a small amount of care and kindness might have developed them into comparatively useful citizens.

GOOD services do not always meet the recognition they should, but when they do, not only gratification is afforded the recipient, but workers of all kinds receive encouragement. It is pleasant to learn that the Dundee Infirmary, and not only the infirmary, but the medical profession of Dundee and the neighbourhood, have shown ample proof of the appreciation of Dr. M'Cosh's services. Dr. M'Cosh has been Superintendent of the Dundee Infirmary during seventeen years. During that time his efforts have been unceasing to render the institution efficient in every respect, and with that success that doubtless to himself is alone reward sufficient for his labours. It is none the less satisfactory that he has won the admiration of his fellow-workers, and the testimonial of a valuable gold signet ring, accompanied by an illuminated address on vellum, records for all time good work on the one hand and good will on the other. The address runs as follows: "Presented to R. Neaves M'Cosh, Esq., M.A., M.D., together with a gold signet ring, in token of the affectionate esteem and regard in which he is held by the whole medical, and nursing, and general staff of the Dundee Royal Infirmary, and in grateful recognition of his uniformly kind and courteous bearing towards all who have been associated with him in his work, on the occasion of his retiring from the office of Superintendent after seventeen years' service.

